# SYBR Green Real-Time PCR-RFLP Assay Targeting the *Plasmodium* Cytochrome B Gene – A Highly Sensitive Molecular Tool for Malaria Parasite Detection and Species Determination

**DOI:** 10.1371/journal.pone.0120210

**Published:** 2015-03-16

**Authors:** Weiping Xu, Ulrika Morris, Berit Aydin-Schmidt, Mwinyi I. Msellem, Delér Shakely, Max Petzold, Anders Björkman, Andreas Mårtensson

**Affiliations:** 1 Department of Microbiology, Tumor and Cell Biology, Division of Malaria Research, Karolinska Institutet, Stockholm, Sweden; 2 Zanzibar Malaria Elimination Programme, Zanzibar, Tanzania; 3 Center for Applied Biostatistics, Sahlgrenska Academy, University of Gothenburg, Gothenburg, Sweden; 4 Department of Public Health Sciences, Division of Global Health (IHCAR), Karolinska Institutet, Stockholm, Sweden; 5 Centre for Clinical Research Sörmland, Uppsala University, Uppsala, Sweden; Université Pierre et Marie Curie, FRANCE

## Abstract

A prerequisite for reliable detection of low-density *Plasmodium* infections in malaria pre-elimination settings is the availability of ultra-sensitive and high-throughput molecular tools. We developed a SYBR Green real-time PCR restriction fragment length polymorphism assay (cytb-qPCR) targeting the cytochrome b gene of the four major human *Plasmodium* species (*P*. *falciparum*, *P*. *vivax*, *P*. *malariae*, and *P*. *ovale*) for parasite detection and species determination with DNA extracted from dried blood spots collected on filter paper. The performance of cytb-qPCR was first compared against four reference PCR methods using serially diluted *Plasmodium* samples. The detection limit of the cytb-qPCR was 1 parasite/μl (p/μl) for *P*. *falciparum* and *P*. *ovale*, and 2 p/μl for *P*. *vivax* and *P*. *malariae*, while the reference PCRs had detection limits of 0.5–10 p/μl. The ability of the PCR methods to detect low-density *Plasmodium* infections was then assessed using 2977 filter paper samples collected during a cross-sectional survey in Zanzibar, a malaria pre-elimination setting in sub-Saharan Africa. Field samples were defined as ‘final positive’ if positive in at least two of the five PCR methods. Cytb-qPCR preformed equal to or better than the reference PCRs with a sensitivity of 100% (65/65; 95%CI 94.5–100%) and a specificity of 99.9% (2910/2912; 95%CI 99.7–100%) when compared against ‘final positive’ samples. The results indicate that the cytb-qPCR may represent an opportunity for improved molecular surveillance of low-density *Plasmodium* infections in malaria pre-elimination settings.

## Introduction

Increased malaria control efforts have resulted in a 25% reduction in the global malaria incidence over the past decade [1–3]. Declining transmission intensities, in areas of successful malaria control, have resulted in a relative increase in the proportion of low-density infections that fall below the detection level of both rapid diagnostic tests and microscopy [1,4,5]. To ensure reliable and timely detection of these low-density infections, ultra-sensitive and high throughput molecular tools are needed, in order to achieve and maintain progress towards malaria elimination [5–7].

Dried blood spots on filter paper are often collected in community-based cross sectional surveys as it is a convenient method for storing samples in the field [8]. The DNA is relatively well preserved, and the samples are easy to transport to laboratory settings without the need of a cold chain. Many PCR methods for malaria case detection have been developed for the diagnosis of symptomatic, often high-density malaria infections, and evaluated in well-equipped laboratory settings where DNA was extracted from 200 μL of whole blood using commercially available kits [9–15]. However, as malaria prevalence declines, sample sizes in cross-sectional surveys increase requiring relatively inexpensive and simple DNA extraction methods.

We have developed and herein described a new SYBR Green real-time PCR (cytb-qPCR) targeting the cytochrome b (cytb) gene of the four major human *Plasmodium* species (*P*. *falciparum*, *P*. *vivax*, *P*. *malariae*, and *P*. *ovale*), followed by *Plasmodium* species (spp.) determination using restriction fragment length polymorphism (RFLP) analysis of PCR-amplified fragments. The cytb-qPCR was primarily developed for detection of low-density malaria infections, in DNA extracted by the Chelex boiling method from samples collected on filter paper.

The performance of the cytb-qPCR was first compared against four highly referenced PCR methods for malaria detection [11,13,16–19]. The ability of the PCR methods to detect low-density *Plasmodium* infections was then assessed using 2977 filter paper blood samples collected during a cross-sectional survey conducted 2011 in Zanzibar, a malaria pre-elimination setting in sub-Saharan Africa.

## Materials and Methods

### Evaluation of primer and probe binding sites by sequence alignment

Sequencer version 5.0 (Gene Codes Co, Arbor, MI) and ClustalW2 software (http://www.ebi.ac.uk/Tools/msa/clustalw2/) were used to align the DNA sequences, primers and probes. The results were presented in area charts for evaluation of PCR primer and probe binding sites.

There are five copies of the 18S rRNA gene in the *P*. *falciparum* and *P*. *vivax* genomes [20], with diverse sequences distributed on five chromosomes. Sequences for all five copies were obtained from the PlasmoDB database version 9.3 (http://plasmodb.org/plasmo/) with accession numbers PF3D7_1148600, 1371000, 0112300, 0531600, 0725600 and PVX_079693, 096002, 097020, 088869, 110844 for *P*. *falciparum* and *P*. *vivax*, respectively. Genome sequencing data for *P*. *malariae* and *P*. *ovale* were not available in the open access database, so their 18S rRNA gene sequences were not aligned.

Unlike the diverse sequences of the 18S rRNA gene, *P*. *falciparum*, *P*. *vivax*, *P*. *malariae* and *P*. *ovale* have a single cytb gene in the mitochondrial DNA, which occurs in multiple identical copies depending on the number of mitochondria per parasite. The sequences of the cytb gene from each of the four major human *Plasmodium* spp. were obtained from the NCBI database (http://www.ncbi.nlm.nih.gov/) with the GenBank accession numbers NC_002375 (*P*. *falciparum*), AY598035 (*P*. *vivax*), AB354570 (*P*. *malariae*), and AB182497.1 (*P*. *ovale*).

### Samples

Dilution series were prepared from *in vitro* cultured samples of *P*. *falciparum* 3D7 and one blood sample each of *P*. *vivax*, *P*. *malariae* and *P*. *ovale* from patients diagnosed and treated at the Karolinska University Hospital, Stockholm, Sweden. *P*. *falciparum* 3D7 cultures were synchronized to the ring stage. Giemsa-stained thin blood smears were prepared and examined under oil immersion (×100 magnification). The number of visualized ring-stage parasites was estimated against 10,000 red blood cells by an expert microscopist where after the hematocrit was set to 50%. Parasite densities of *P*. *vivax*, *P*. *malariae*, and *P*. *ovale* were determined by Giemsa-stained thick blood smears. The numbers of parasites were counted against 500 white blood cells and the parasite density, in parasites/μl (p/μL), was determined assuming 8000 white blood cells/μl of blood. For *P*. *vivax*, the parasite density was also determined against 10,000 red blood cells in a thin blood smear. Tenfold dilution series were prepared, in fresh uninfected whole blood, from 10000–1 p/μL for *P*. *falciparum*, *P*. *vivax*, and *P*. *malariae* and 2000–2 p/μl for *P*. *ovale* (due to lower parasite density). In addition low-density dilution series of 20, 10, 5, 2, 1 and 0.5 p/μl were prepared for each species. Approximately 30 μl of each dilution was spotted onto Whatman 3MM filter paper (GE Healthcare, Buckinghamshire, UK) and air-dried.

Field samples were collected from individuals participating in a community based cross-sectional survey conducted in Zanzibar, 2011. A total of 2977 blood spots were collected on filter paper (Whatman 3MM), air dried, individually packaged in plastic envelopes with desiccants, and shipped to Sweden for molecular analysis.

Malaria negative samples consisted of whole blood collected on filter paper from individuals residing in Sweden with no history of malaria exposure.

### DNA extraction

DNA was extracted from the dry blood spots on filter paper using the Chelex boiling method as described by Wooden *et al*. [21] with minor modifications. Briefly, a filter paper disk of Ø 3 mm with blood saturated on both sides (≈ 3–5 μl blood) was cut into a 1.5 ml safe-lock tube with a manual paper puncher. The paper disk was suspended in 1 ml 0.5% saponin (Sigma-Aldrich, St Louis, MO) in phosphate buffered saline (PBS; Gibco, Paisley, UK) at 4°C overnight, and then washed in 1 ml PBS at 4°C for 30 min. The wet filter paper was subsequently incubated at 95°C for 10 min in 100 μl 10% Chelex in molecular grade water (Sigma-Aldrich), and centrifuged at 10000 g for 2 min. The supernatant containing genomic DNA was obtained and stored at −20°C. The dilution series were extracted in quadruplicate and the field samples once individually.

### Cytb-qPCR assay

The cytb-qPCR primers were designed using web-based software Primer3 v.0.4.0 (http://frodo.wi.mit.edu/), targeting a conserved area in the cytb gene of the four major human *Plasmodium* spp. The sequences of the forward and reverse primers were 5’-TGG TAG CAC AAA TCC TTT AGG G-3’ and 5’-TGG TAA TTG ACA TCC AAT CC-3’, respectively. The PCR reaction was carried out in a final volume of 20 μl, containing 5 μl of extracted DNA, 0.25 μM of each primer, and 1 × iTaq Universal SYBR Green Supermix (Bio-Rad, Hercules, CA). Real-time PCR was performed in an ABI Prism 7000 system (Applied Biosystems, Foster City, CA) as follows: 95°C for 4 min; 40 cycles of 95°C for 15 s, 60°C for 1.5 min; 72°C for 5 min; and melting curve acquisition. The fluorescence signal was obtained at the end of each 60°C step, and continuously during the melting curve acquisition. Cytb-qPCR positivity was confirmed by gel electrophoresis of qPCR products on a 1.5% agarose gel stained with GelRed (Biotium Inc., Hayward, CA). Positive and negative controls were included in each run.

*Plasmodium* spp. was determined by RFLP on all positive cytb-qPCR products using single enzyme digestion with either one of the following four enzymes: FspBI (Thermo Fisher, Waltham, MA), AluI (NEB, New England Biolabs, Hitchin, UK), HpyCH4V (NEB), or Csp6I (Thermo Fisher). The RFLP reaction was conducted in a 20 μl volume with 5 μl of qPCR product and 5 units of respective enzyme in 1 × reaction buffer, following the manufactures instructions. After overnight digestion at 37°C, RFLP products were run on 2% agarose gel stained with GelRed (Biotium), and documented with a Gel-doc system (Bio-Rad). DNA from reference species were included as a digestion control, allowing for species determination by direct comparison of RFLP patterns of unknown sample with the reference. In a few cases, where the cytb-qPCR products were of correct size but showed unique RFLP patterns compared to the reference species, *Plasmodium* spp. was determined by DNA sequencing performed by GATC Biotech AG (Constance, Germany).

### Primer specificity and cytb-qPCR efficiency

The cytb-qPCR primer amplification specificity was tested by gel electrophoresis of qPCR products on a 1.5% agarose gel stained with GelRed (Biotium), by Primer-blast (http://www.ncbi.nlm.nih.gov/tools/primer-blast/) against the NCBI nucleotide and chromosome database, and on DNA extracted from malaria negative whole blood samples. The cytb-qPCR efficiency was tested on the tenfold dilution series. The dilutions series were extracted in quadruplicate and the cytb-qPCR was performed in duplicate, i.e. a total of eight PCR replicates at each dilution point. The cytb-qPCR efficiency (E) and coefficient of determination (R^2^) of the standard curves were calculated for each species.

### Reference PCR methods

1. Snounou *et al*. [18], established one of the earliest nested PCR methods targeting the 18S rRNA sequences of *Plasmodium* spp. (18S-nPCR). This method allows for *Plasmodium* spp. detection using pan-*Plasmodium* primers, and *Plasmodium* spp. determination (in pan-*Plasmodium* PCR positive samples) using species-specific nested primers in four additional individual PCRs.

2. Rougemont *et al*. [13], developed a probe-based qPCR method targeting the 18S rRNA sequences of *Plasmodium* spp. (18S-qPCR-R). The method allows *Plasmodium* detection using pan-*Plasmodium* primers and probe. PCR positivity was defined as quantification cycle (Cq) values below 40. The method also allows for species determination by additional qPCRs using species-specific probes, but was not evaluated in this study due to the experienced low specificity of the pan-*Plasmodium* 18S-qPCR-R (see results).

3. Kamau *et al*. [11], also developed a probe-based qPCR method targeting the 18S rRNA sequences of *Plasmodium* spp. (18S-qPCR-K). PCR positivity was defined as Cq values below 40. This method is designed for pan-*Plasmodium* detection only, and does not allow for species determination.

4. Steenkeste *et al*. [19], established a nested PCR targeting the cytb gene of the four major human *Plasmodium* spp. (cytb-nPCR). This method was further modified by Hsiang *et al*. [22] for use of RFLP analysis with AluI enzyme for species determination instead of the original dot-blot or sequencing assay.

The reference PCRs were performed according to the original protocols, the only deviation being the quantity of starting DNA template which was kept at 5 μl of DNA for all PCRs. Positive and negative controls were included in all PCR runs.

### Parasite detection limits

The detection limits of the cytb-qPCR and four reference PCRs were determined using pan-*Plasmodium* primers and probes. Detection limits were determined using DNA extracted in quadruplicate from the low-density dilution series of 20, 10, 5, 2, 1, and 0.5 p/μl prepared for each species together with a malaria negative control. The detection limits for each species were defined as the lowest consecutive dilution level that was PCR positive in at least three out of four replicates.

### *Plasmodium* detection and species determination in field samples

DNA from the 2977 field samples were extracted individually, and screened for *Plasmodium* spp. with the four reference PCRs and the cytb-qPCR, using pan-*Plasmodium* primers and probes.

*Plasmodium* spp. were determined in three out of the five PCRs. For the cytb-qPCR positive samples, *Plasmodium* spp. was determined by FspBI and AluI RFLP assays. For the 18S-nPCR and cytb-nPCR methods, *Plasmodium* spp. were determined as described above by individual nested PCRs and AluI RFLP, respectively. The results for the three PCRs were combined to give the ‘final species’ for each sample, for example if two PCRs detected different species then the sample was determined as a mixed infection in the ‘final species’. Species determination with species-specific probes described in the 18S-qPCR-R method was not evaluated in this study due to the experienced low specificity of the pan-*Plasmodium* 18S-qPCR-R (see results). The 18S-qPCR-K method is designed for pan-*Plasmodium* detection only, and does not allow for species determination.

### Statistical analyses

To determine the cytb-qPCR efficiency, linear regression assay was used to analyse the standard curves derived from tenfold dilution series of *P*. *falciparum*, *P*. *vivax*, *P*. *malariae*, and *P*. *ovale* samples. Due to the large difference in PCR positivity rates between the methods, and in order to minimize the influence of possible false positive PCR results, field samples were defined as ‘final positive’ if positive in at least two of the five PCR methods. The difference in the proportion of positive samples between individual PCR conditions and the ‘final positive’ samples was compared by McNemar’s exact test for paired data. The level of agreement between the PCR conditions and the ‘final positive’ samples was assessed by kappa analysis (κ). The sensitivity and specificity for each PCR method was calculated with 95% confidence intervals (95% CI). To minimize the influence of cytb-qPCR results on the ‘final positive’ samples, statistical analyses were repeated after ‘final positive’ samples were re-defined as positivity in at least two out of the four reference PCR methods. For the species determination, kappa analysis was conducted to determine the agreement between the 18S-nPCR, cytb-nPCR, cytb-qPCR methods, and the ‘final species’ outcome. Statistical analyses were conducted using STATA v.12 software (Stata Corp, Texas, USA). Statistical significance was defined as *p* < 0.05.

### Ethical considerations

The study was approved by the ethical committee in Zanzibar (ZAMREC/001/June/011) and the Regional Ethics Committee in Stockholm, Sweden (2009/387-31). All participants in the cross-sectional survey provided written informed consent prior to blood sampling; in case of children a written consent was provided from caretakers.

## Results

### Sequence alignments

**Fig 1:**
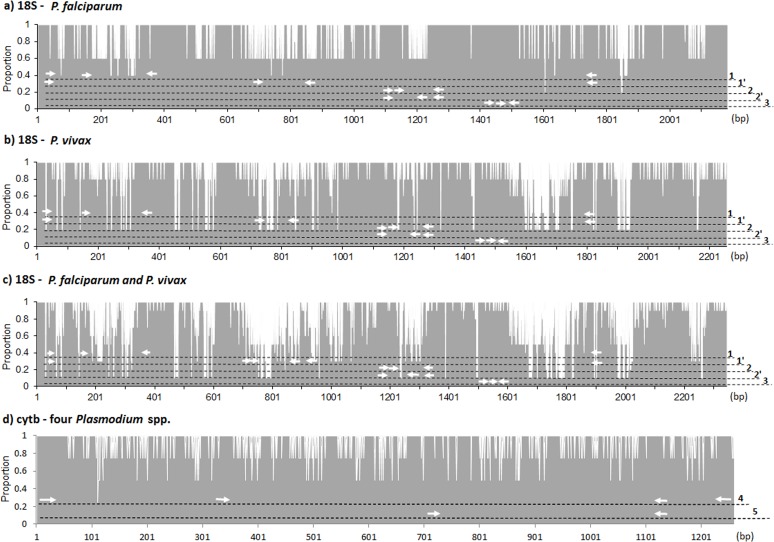
Area chart showing the sequence alignments of target genes, primers and probes. a) Five copies of the 18S rRNA gene in the *P*. *falciparum* genome. b) Five copies of the 18S rRNA gene in the *P*. *vivax* genome. c) Combined ten copies of the 18S rRNA gene in the *P*. *falciparum* and *P*. *vivax* genomes. d) The cytb genes of *P*. *falciparum*, *P*. *vivax*, *P*. *malariae*, and *P*. *ovale*. Identical sequences are shown in grey in the chart area. Nucleotide base pair position is shown on the X-axis and the proportion of sequences in consensus is shown on the Y-axis. Primer and probe positions of respective PCRs are indicated using white arrows along the dashed lines (1, 18S-nPCR pan-*Plasmodium* primers; 1’, 18S-nPCR species-specific primers; 2, 18S-qPCR-R pan-*Plasmodium* primers and probe; 2’, 18S-qPCR-R species-specific primers and probes; 3, 18S-qPCR-K; 4, cytb-nPCR; and 5, cytb-qPCR).

Sequence alignments of the *P*. *falciparum* and *P*. *vivax* 18S rRNA genes and PCR primer and probe binding sites are shown in Fig. 1A-C. The pan-*Plasmodium* primer and probe binding sites of the 18S-nPCR, 18S-qPCR-R and 18S-qPCR-K are located in conserved areas targeting all five copies of the 18S rRNA genes in both the *P*. *falciparum* and *P*. *vivax* genomes. The species-specific primers and probe of the 18S-nPCR and 18S-qPCR-R are located in hyper-variable regions targeting two to three copies of 18S rRNA genes.

Sequence alignment of the *Plasmodium* cytb genes and PCR primer binding sites are shown in Fig. 1D. Primers of cytb-nPCR and cytb-qPCR are located in the conserved regions targeting four major human *Plasmodium* spp., framing plural hyper-variable sequences allowing for species determination by RFLP assay or sequencing.

### Cytb-qPCR primer specificity and species determination

**Fig 2:**
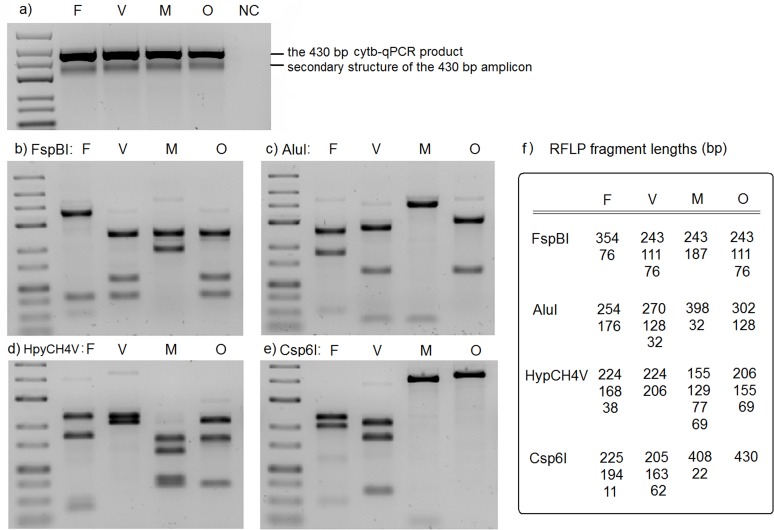
The cytb-qPCR products and RFLP assays for species determination. a) The 430 bp cytb-qPCR products for each species. b-e) Four RFLP assays using FspBI, AluI, HpyCH4V and Csp6I restriction enzymes for *Plasmodium* species determination. f) Schematic diagram showing the RFLP product lengths. a-e) Lane 1, GeneRuler low range DNA ladder (Thermo Fisher); F, *P*. *falciparum*; V, *P*. *vivax*; M, *P*. *malariae*; O, *P*.*ovale*; NC, negative control.

Gel electrophoresis of the amplified qPCR products showed a strong band at 430 bp. A weaker secondary band was sometimes seen below the main band (Fig. 2A). Gel extraction and sequencing of both bands showed identical sequences to the reference sequence, indicating the weaker band to be a secondary structure of the main PCR product rather than unspecific amplification. Primer-blast analysis of the cytb-qPCR primers against the NCBI nucleotide and chromosome database found no target templates other than *Plasmodium* spp. Primer specificity was also tested on the malaria negative samples, all of which were negative by cytb-qPCR. The melting temperatures for each species in the cytb-qPCR were 74.5°C for *P*. *falciparum*, 74.0°C for *P*. *vivax*, 73.5°C for *P*. *malariae* and 74.0°C for *P*. *ovale*. The limited differences preclude melting temperatures to be used for species determination. Instead four RFLP assays were used, singly or in parallel depending on the parasite species complexity, to distinguish *Plasmodium* spp. (Fig. 2B-F).

### Amplification efficiency and reproducibility of the cytb-qPCR

**Fig 3:**
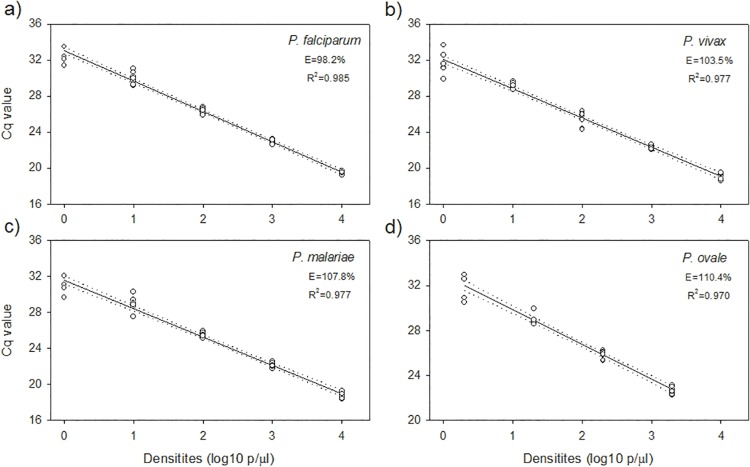
The cytb-qPCR standard curves derived from tenfold dilution series of *P*. *falciparum*, *P*. *vivax*, *P*. *malariae*, and *P*. *ovale* samples. The cytb-qPCR efficiency (E) and coefficient of determination (R^2^) of the standard curves were calculated for each species. The cytb qPCR was conducted in eight replicates (4 extractions × 2 cytb-qPCRs). Dashed lines represent 95% CI.

The amplification efficiency of the cytb-qPCR was 98.2–110.4%, with R^2^ values between 0.97–0.99, showing a linear correlation (*p* < 0.01) between the Cq values and log 10 parasite densities (Fig. 3). For parasite densities ≥10 p/μl, the cytb-qPCR had high PCR reproducibility, with eight out of eight PCR replicates being positive. For parasite densities at 1 or 2 p/μl, the cytb-qPCR was reproducible in four to six out of the eight replicates.

### Parasite detection limits

**Fig 4:**
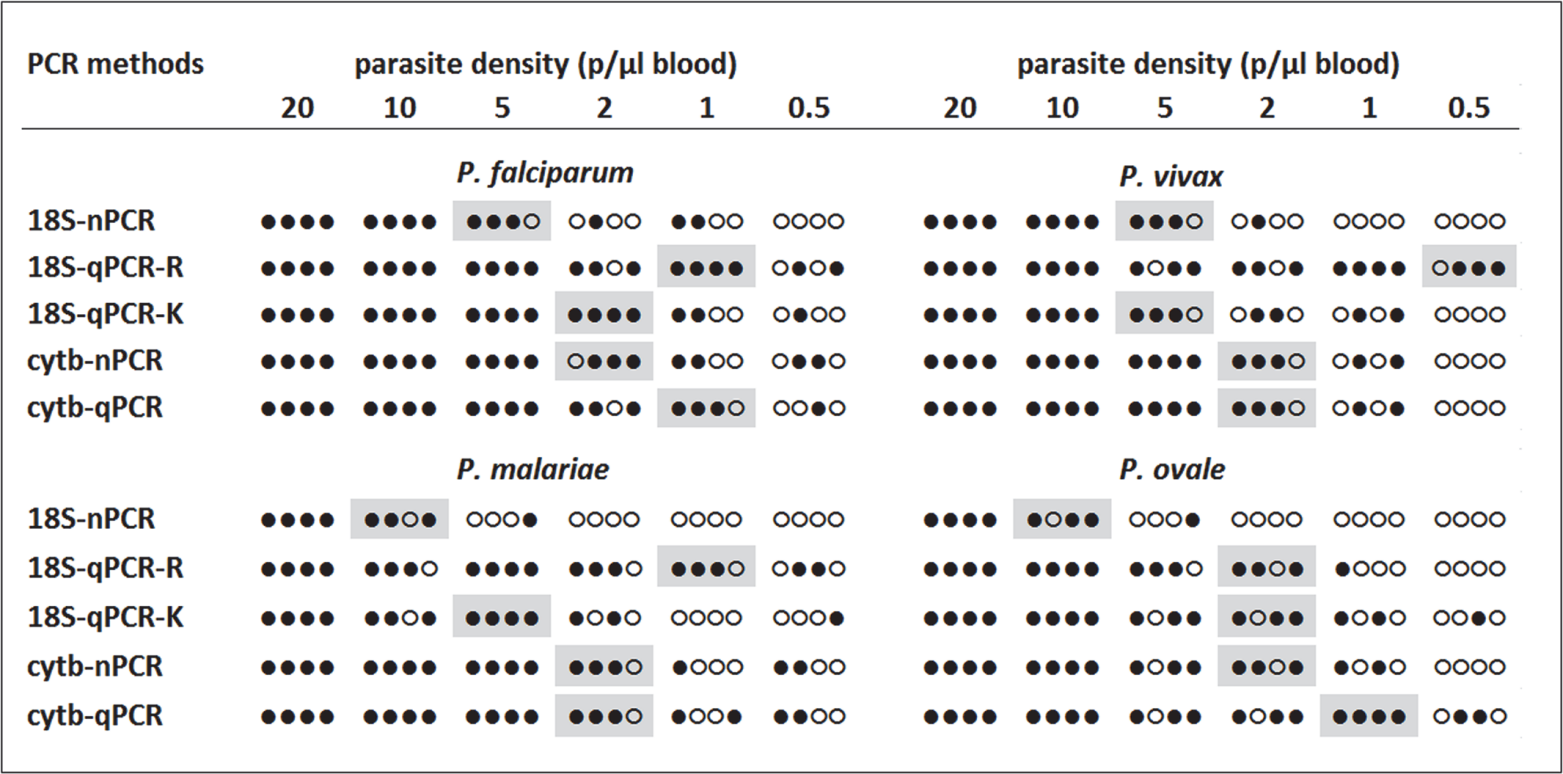
Parasite detection limits for each PCR method. Solid dots represent PCR positive and hollow dots represent PCR negative samples. Detection limits are shaded in grey.

The parasite detection limits for the cytb-qPCR were 1 p/μl for *P*. *falciparum*, 2 p/μl for *P*. *vivax*, 2 p/μl for *P*. *malariae* and 1 p/μl for *P*. *ovale* (Fig. 4). The corresponding detection limits for the reference methods were 5–10 p/μl for the 18S-nPCR, 0.5–2 p/μl for the 18S-qPCR-R, 2–5 p/μl for the 18S-qPCR-K, and 2 p/μl for the cytb-nPCR (Fig. 4).

### *Plasmodium* detection in field samples

**Fig 5:**
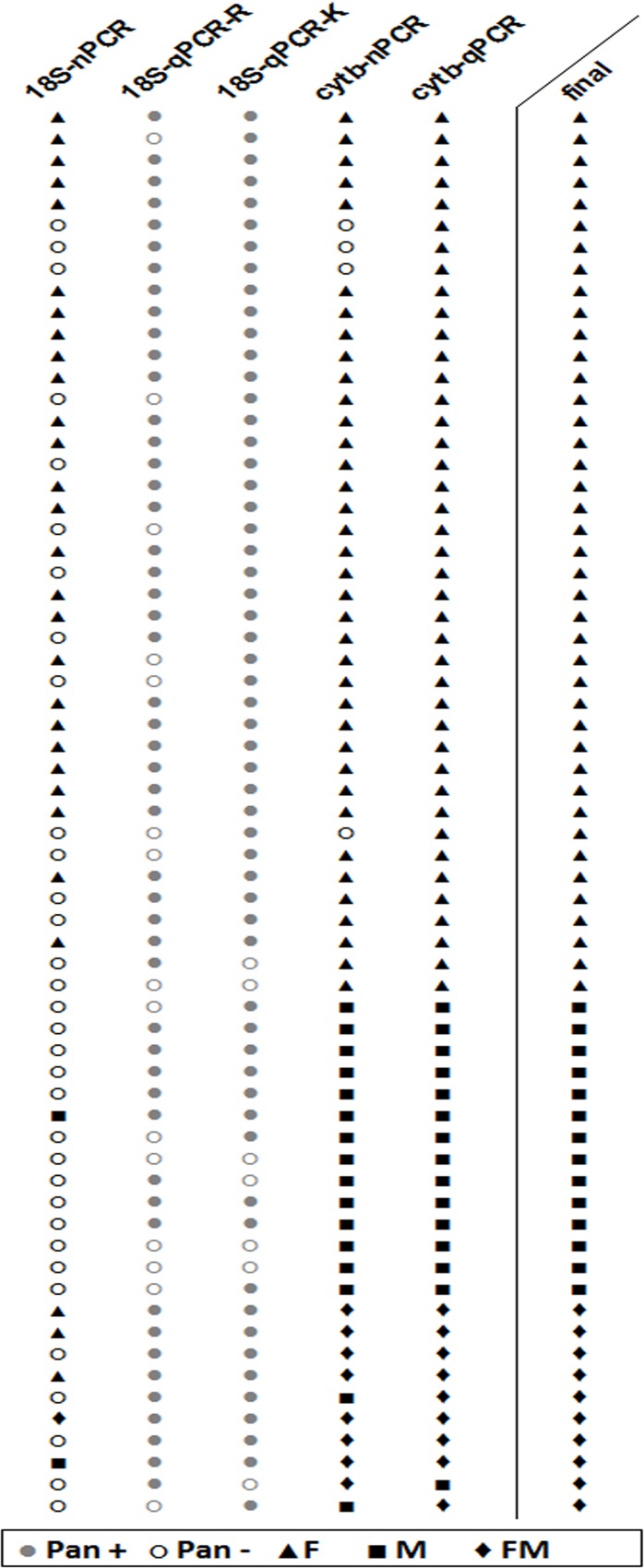
PCR and species results for the 65 ‘final positive’ field samples. Each row represents one sample. Pan, *Plasmodium spp*.; F, *P*. *falciparum*; M, *P*. *malariae*; FM, *P*. *falciparum* and *P*. *malariae* mixed infection; +, positive; −, negative.

The proportion of field samples positive by pan-*Plasmodium* PCR ranged from 1.1% to 14.8% in the different PCR methods (Table 1). In total, 65 samples (2.2%; CI 95% 1.6–2.7) were determined as ‘final positive’ by being PCR positive in at least two out of the five methods (Fig. 5). There was high level of agreement (κ = 0.86–0.98), high sensitivity (89.2–100%) and high specificity (99.6–100%) between the 18S-qPCR-K, cytb-nPCR, cytb-qPCR and the ‘final positive’ samples. The 18s-nPCR found a significantly lower number of PCR positives (*p* < 0.01) resulting in a lower sensitivity (49.2%; CI 95% 36.6–61.9). The 18S-qPCR-R had a significantly higher number of PCR positives (*p* < 0.01) resulting in a lower specificity (86.6%; 95% CI 85.3–87.8).

**Table 1:** Proportion of pan-*Plasmodium* positive samples detected by each PCR compared with the ‘final positive’ samples.

PCR methods	Positivity ^a^	*P* ^b^	Kappa ^b^	Sensitivity ^b^	Specificity ^b^
	n (%; 95% CI)			% (95% CI)	% (95% CI)
**18S-nPCR**	32 (1.1; 0.7–1.5)	<0.01	0.65	49.2 (36.6–61.9)	100 (99.9–100)
**18S-qPCR-R**	441 (14.8; 13.6–16.1)	<0.01	0.17	76.9 (64.8–86.5)	86.6 (85.3–87.8)
**18S-qPCR-K**	69 (2.3; 1.8–2.9)	0.48	0.86	89.2 (79.1–95.6)	99.6 (99.3–99.8)
**cytb-nPCR**	61 (2.0; 1.6–2.6)	0.13	0.97	93.8 (85.0–98.3)	100 (99.9–100)
**cytb-qPCR**	67 (2.3; 1.7–2.8)	0.50	0.98	100 (94.5–100)	99.9 (99.8–100)
**‘final positive'**	65 (2.2; 1.7–2.8)	-	-	-	-

^a^ Proportion of samples positive in the pan-*Plasmodium* screening of field samples (N = 2977).

^b^ The *p* value, kappa value, sensitivity and specificity were calculated by comparing individual PCRs with the ‘final positive’ samples defined as positivity in at least two of the five PCR methods.

A total of 64 samples were determined as ‘final positive’ when ‘final positive’ was re-defined as positivity in at least two out of the four reference PCRs. However, this did not change the *p* value (0.25), κ value (0.98), sensitivity (100%; 95% CI, 94.4–100%) or specificity (99.9%; 95% CI, 99.7–100%) estimates for the cytb-qPCR considerably.

### Species determination in field samples

*Plasmodium* spp. was determined for the 65 ‘final positive’ samples by the 18S-nPCR, cytb-nPCR and cytb-qPCR. Overall 41 *P*. *falciparum* mono-infections, 14 *P*. *malariae* mono-infections, and 10 *P*. *falciparum* and *P*. *malariae* mixed infections were detected; no other species were found (Fig. 5, Table 2). Kappa analyses showed high agreement between the cytb-nPCR and cytb-qPCR (κ = 0.81) as well as with the ‘final species’ outcome (κ = 0.84–0.97). The 18S-nPCR had low agreement with the ‘final species’ outcome (κ = 0.20).

**Table 2:** *Plasmodium* species detected by three PCR methods compared with the ‘final species’.

PCR methods	*P*. *falciparum*	*P*. *malariae*	Mixed ^b^	Sum	Kappa ^c^
**18S-Npcr**	29	2	1	32	0.20
**cytb-nPCR**	37	16	8	61	0.84
**cytb-qPCR**	41	15	9	65	0.97
**‘final species’** ^a^	41	14	10	65	-

^a^ The ‘final species’ is the combined species results of the three PCRs.

^b^ Mixed means *P*. *falciparum* and *P*. *malariae* mixed infection.

^c^ The kappa values were calculated by comparing individual PCRs with the ‘final species’.

## Discussion

We developed a SYBR Green based qPCR targeting the cytochrome b gene in the four main *Plasmodium* spp. that infect humans for detection of low-density infections in blood samples collected on filter paper. The performance of the cytb-qPCR was compared with four reference PCR methods [11,13,18,19]. The ability of the five PCR methods for detection of low-density *Plasmodium* infections in a malaria pre-elimination setting was assessed using 2977 samples collected from individuals participating in a community based cross-sectional survey in Zanzibar. Overall, the cytb-qPCR performed equal to, or better than the reference PCR methods. It had a parasite detection limit of 1–2 p/μl, robust PCR amplification efficiency and could reliably determine *Plasmodium* spp. by RFLP.

Many PCR methods for malaria detection have been developed for DNA extracted from 200 μL of whole blood using commercially available kits [9–15]. In contrast, this study evaluated the performance of the PCRs on DNA extracted by Chelex, a relatively inexpensive method of DNA extraction [23], from one 3 mm punch of filter paper containing approximately 3–5 μL blood (one fortieth of the biological material used in whole blood extraction). The detection limits we obtained with the cytb-qPCR (1–2 p/μL), with one fortieth of the biological material, are therefore comparable with the previously reported detection limits for the 18S-qPCR-K [11] and the cytb-nPCR [19] (0.05 p/μL and 0.075 p/μL, respectively).

A single qPCR for both detection and determination of *Plasmodium* spp. would be ideal for molecular surveillance of low-density infections. Unfortunately, this was not possible with the cytb-qPCR as the high AT-content (73%) of the amplified PCR products resulted in low melting temperatures, which could not be fully distinguished from the melting temperatures of primer dimers, even after optimization of primer concentrations and annealing temperatures (data not shown). The cytb-qPCR therefore requires gel-electrophoresis for confirming PCR positive samples and RFLP for species determination. Although this extends the sample processing time, we believe there are still some advantages of the cytb-qPCR assay over the reference methods. The qPCR-RFLP assay is highly sensitive requiring only a single round of PCR, halving the time compared to nested PCRs. Furthermore, although the reference qPCR assays require no gels or RFLP, neither provided optimal methods for species determination which is of importance in molecular surveillance of malaria in pre-elimination settings such as Zanzibar [24].

Large discrepancies in PCR positivity rates were observed between the five methods when screening the 2977 field samples. We applied a conservative approach and compared the PCR methods against ‘final positive’ samples defined as PCR positivity in at least two of the five methods. Using this approach the 18S-qPCR-K, cytb-nPCR and cytb-qPCR showed high sensitivity and specificity, consistent with previously published comparative evaluations of PCRs [12,22,25]. A small difference in the PCR outcome is to be expected, since most of these infections are likely to consist of low-density parasitaemias close to the detection limits of the PCRs [24].

Differences in the PCR sensitivities may be attributed to variations in the PCR product size, target gene copy number as well as PCR primer and probe binding sites. The size of the first PCR product of the 18S-nPCR is 1726 bp, which is substantially larger than the 100 bp, 815 bp and 430 bp products of the 18S-qPCR-K, cytb-nPCR and cytb-qPCRs, respectively. DNA extraction by Chelex involves boiling at 95°C; this may cause fragmentation of genomic DNA, resulting in reduced PCR efficiency of larger PCR products and consequently reduced PCR sensitivity [26].

Although the pan-*Plasmodium* primers and probes targeted all five copies of the 18S rRNA genes in *P*. *falciparum* and *P*. *vivax*, the cytb PCRs are likely to have a much higher target copy number, since a single *P*. *falciparum* parasite consists of approximately 20–150 mitochondria, each carrying a single, identical cytb gene [27,28]. Furthermore, mitochondrial DNA may be better preserved than genomic DNA [19] providing an added value of targeting cytb rather than 18s rRNA genes. The superiority of cytb over 18S rRNA as a target for PCR has also been shown in the detection of malaria in mosquitoes [29] and well as in human blood, urine and saliva samples [30].

Importantly, PCR primers and probes play an important role in PCR efficiency and specificity. The 18S-qPCR-R showed a significantly higher PCR positive rate and lower specificity than the other PCRs, probably due to unspecific amplification and/or probe degradation at high quantification cycle numbers. Rougemont *et al*. [13] describe in their original report the hybridization of the 18S-qPCR-R pan-*Plasmodium* probe with several homologous eukaryotes including *Aspergillus*, *Toxoplasma*, *Neospora*, and *Pneumocystis spp*. Even though the 18S-qPCR-R showed a high analytical sensitivity, the low specificity of this test reduces its usefulness for screening of low-density *Plasmodium* infections in field samples.

Furthermore, there may be differences in the sensitivities of the pan-*Plasmodium* and species-specific PCRs. As seen in the sequence alignments in Fig. 1 there is substantial variation between the five copies of the 18S rRNA genes. The species-specific primers of the 18S-nPCR (and primers and probes of the 18S-qPCR-R, although not evaluated in this study) target hyper-variable regions in the 18S rRNA gene. This may result in the amplification of only two or three out of the five 18S rRNA copies further reducing the sensitivity of the species-specific PCRs.

Limitations of this study include low repeatability of PCR-based results for detection of low-density infections with parasite densities approaching the PCR detection limits [31]. Reproducibility of results from DNA extraction to PCR can also be influenced by non-homogenous distribution of parasites throughout dried blood spots [25], resulting in varying parasite densities in different DNA extractions. Secondly, single nucleotide polymorphisms in the target gene may result in unique RFLP patterns of cytb-qPCR amplified products. This was observed in less than 5% of the samples and in these cases sequencing of the PCR product was required to determine the species. Thirdly, due to their close phylogenetic relationship [32,33] the RFLP assay cannot distinguish between *P*. *vivax* and *P*. *knowlesi* nor between the two cryptic species of *P*. *ovale* (*P*. *ovale curtisi* and *P*. *ovale wallikeri*).

Finally, there are no optimal statistical tools available for comparisons between multiple methods, which needs to be taken into consideration when interpreting our results. Microscopy remains the golden standard for malaria diagnosis, however as malaria prevalence decreases microscopy becomes a less useful tool as the relative proportion of submicroscopic infections increases [5]. Microscopy is not a suitable gold standard in low-transmission settings, and was not conducted during the 2011 cross-sectional survey. Instead, we compared the PCR methods against ‘final positive’ samples defined as PCR positivity in at least two of the five methods. Despite being a conservative approach this method may have inherent limitations considering that the ‘final positives’ are closely related to the cytb-qPCR positive samples. To minimize the influence of cytb-qPCR results on the ‘final positive’ samples, statistical analyses were repeated after ‘final positive’ samples were re-defined as positivity in at least two out of the four reference PCR conditions. Repeated analysis did not change the agreement, sensitivity and specificity considerably.

## Conclusions

The cytb-qPCR was primarily developed for detection of low-density malaria infections, in DNA extracted by the Chelex boiling method from samples collected on filter paper. Overall, the cytb-qPCR assay performed equal to, or better than the four reference PCR methods for parasite detection and species determination. The results indicate that the cytb-qPCR may represent an opportunity for improved molecular surveillance of low-density *Plasmodium* infections in malaria pre-elimination settings.
